# A study on the difference between two types of mountaineering outerwear in a cold, wet and windy environment

**DOI:** 10.1186/2046-7648-4-S1-A91

**Published:** 2015-09-14

**Authors:** Li-Chu Wang, Hsien-Chen Chou, Gwo-Tsuen Jou

**Affiliations:** 1 Department of Testing and Certification, Taiwan Textile Research Institute, Taiwan, Republic of China

## Introduction

For mountaineering, the outer or protective layer of clothing system is especially important for extremely environment. Generally, a breathable barrier embedded in the layered fabric will be used to prevent rain or snow from outside and let the body moisture diffused into air. There are two types of membrane, one is hydrophobic (HPO) and the other is hydrophilic (HPI). A considerable amount of studies claimed that water vapor transport through HPI polymers is highly influenced by the test conditions [1]; in non-isothermal test, the clothing systems incorporating HPI polymers are improved to greater amounts than those incorporating microporous polymers [2]; some experimental results further point out that the water vapor transfer rate of porous polyurethane laminated fabric was greater under isothermal conditions whilst the water vapor transfer rate of HPO laminated fabrics was greater under non-isothermal conditions, especially when a fabric contains more condensation [3]; and, with the use of hot plate and sweating arm system, an EMPA study showed the hydrophilicity and condensation have little effect on effective water vapor resistance of multilayer textile combination in 20 °C but become larger with decreasing outside temperature [4]. In our previous study, the difference between the microstructure of PTFE and the hydrophilicity of PU affects the comfort properties of leisure wearing especially in mild and cool temperature, water vapor resistance testing (Ret), EMPA sweating torso wearing trial simulation, and subjective wearing trials were conducted. The HPO is better in Ret test, though the HPI is slightly better in the non-isothermal state. While the subjective wear trial showed no significant difference [5]. So this extended study aimed to determine the comfort properties by a wear trial in an extremely environment.

## Methods

Two males (age: 21 yrs; height: 170 and 175 cm; mass: 60~65 kg) wore either a HPO or HPI jacket (same as in previous study [5] and with beanie, scarf, and gloves), performed the same protocol involved in 15 minutes of sitting without rain, and 20 minutes of walking (1.5 km.h^-1 ^at 5% gradient) on treadmill, with rain. The environmental conditions were maintained at: 5 ± 1 °C, 50 ± 3 % RH, 150 ± 10 mm.h^-1 ^rainfall, 3 ± 0.5 m.s^-1 ^wind speed. Skin temperature (T_S_), microclimate humidity (H_M_) and temperature (T_M_), IR skin temperature (T_IR_), sweat condensation (S_C_) and perceived comfort were recorded for each participant.

## Results

The results of T_S _and T_M _showed HPI was lower at the beginning and up to 0.7 and 1.3 °C higher at the follow-up stage of the experiment. Thermal perceptions revealed similar trend. H_M _demonstrated similar results of HPO and HPI, while subjective dampness showed 0.5 grade dryer of HPI in walking period. The S_C _weight was 21.2 g and 17.7 g for HPO and HPI respectively. The declines of T_IR _were very similar.

## Discussion

Both for T_S _and T_M_, HPI and HPO crossed each other during walking, and HPI revealed higher warmth keeping ability. S_C _weight was 3.5 g lower and the dampness perception was slightly drier for HPI.

## Conclusion

HPI demonstrated slightly colder at the starting point, but had less sweat condensation in the clothing system, and became warmer after walking for about 10 minutes in a cold, wet and windy environment, suggesting that a better comfort was achieved. However, the subjective perspirations were not significant between HPO and HPI outerwear in terms of comfort property.
